# Portable sauna stimulated-diaphoresis for the treatment of fluid-overload in peritoneal dialysis patients: A pilot study

**DOI:** 10.3389/fmed.2022.887609

**Published:** 2022-09-20

**Authors:** Pablo Maggiani-Aguilera, Jonathan S. Chávez-Iñiguez, Guillermo Navarro-Blackaller, Karla Hernández-Morales, Ariadna Lizbeth Geraldo-Ozuna, Luz Alcantar-Villín, Olivia Montoya-Montoya, Víctor Hugo Luquín-Arellano, Guillermo García-García

**Affiliations:** ^1^Nephrology Department, Civil Hospital of Guadalajara Fray Antonio Alcalde, Guadalajara, Mexico; ^2^University of Guadalajara Health Sciences Center, Guadalajara, Mexico

**Keywords:** peritoneal dialysis, fluid overload, diaphoresis, sauna bath, chronic kidney disease

## Abstract

**Background:**

Fluid overload (FO) is a common problem in patients with peritoneal dialysis (PD), it is associated with adverse outcomes and may persist despite adjustements in PD therapy.

**Objective:**

To evaluate the feasibility and safety of stimulated diaphoresis to reduce FO with the use of a portable sauna bath.

**Methods:**

Open-label pilot study in patients on continuous ambulatory peritoneal dialysis (CAPD) and FO. The primary outcome was the treatment-related adverse events; secondary outcomes were changes in over-hydration (OH), body weight and blood pressure, FO symptoms, and sleep quality. Dialysis prescription and daily data were recorded. The intervention period consisted in a 30-min, 45°C sauna bath, daily for 10 days, using a portable sauna bath.

**Results:**

Fifty-one out of 54 total sauna bath sessions were well tolerated. In three (5.5%) sessions adverse effects were reported: transient dizziness in two cases, and a second-degree skin burn in a patient with advanced diabetic neuropathy. OH (6.3 ± 1.2 L vs. 5.5 ± 1.3 L, *p* = 0.05), body weight (67.7 ± 11.4 vs. 66.8 ± 3.8 kg, *p* = 0.003), diastolic blood pressure (92 ± 13.5 vs. 83 ± 13.3 mmHg, *P* = 0.003) and PSQI score (7.3 ± 3.7 vs. 5.1 ± 3.2, *p* = 0.02) improved significantly between the control and intervention period, respectively.

**Conclusions:**

Stimulated diaphoresis with a portable sauna bath could be a novel, safe, and effective alternative way to reduce FO in CAPD patients. Larger studies are needed to confirm our results.

**Clinical trial registration:**

ClinicalTrials.gov, identifier: NCT03563898.

## Introduction

FO is a common complication in end-stage renal disease (1, 2), and it has been associated to arterial hypertension, left ventricular hypertrophy, malnutrition and inflammation in the end-stage kindey disease (3–7), as well as to all-cause mortality in patients with kidney replacement therapy (KRT) (8–12).

The need of high ultrafiltration rates increases the risk of intradialytic hypotension, cramps, and the impossibility to reach dry weight in the short term (13). In pertioneal dialysis (PD) patients, the use of hypertonic solutions can increase the ultrafiltrate volume, but unfortunately, it may also lead to a more rapid deterioration of peritoneal membrane function, and potentially to the loss of residual renal function (14). Despite improvements in the methods of KRT as well as other techniques in PD patients, the problem of FO persists in the PD population.

Few studies have examined fluid losses through diaphoresis in patients with kidney disease, indicating that fluid loss through diaphoresis can be substantial (15–17). Body sweat is produced by 2–3 million sweat glands to control body temperature (18). Healthy people can secrete up to 4 L of water in sweat in 1 h with appropriate stimuli (15). Exocrine secretion also contains factors that regulate skin flora and reduce the risk of infections (19). Hot water baths performed in two patients induced water losses of 566 ± 160 and 813 ± 62 ml / h, respectively (15). Additionally, improvement of uremic symptoms has been described (17). Our protocol offers a technique not previously used with a portable sauna bath, which is affordable and easy to use at home in patients with PD. We hypothesize that the stimulation of diaphoresis in PD patients with portable sauna can be a safe and effective strategy to reduce FO.

## Materials and methods

### Subjects

This was an open-label, interventional and treatment purpose pilot study, in 9 PD patients with FO from the Hospital Civil de Guadalajara. To evaluate its safety, the sauna therapy was implemented in hospitalized patients, under medical supervision. Inclusion criteria were being on CAPD for at least 3 months, diagnosis of FO (OH > 2 L measured with bioelectrical impedance BCM, Fresenius (R), stable clinical condition, and availability to give informed consent. Exclusion criteria were any cardiovascular event in the last 12 months (acute myocardial infarction, NYHA Heart Failure III-IV, cardiac arrhythmia, cerebral vascular event, unstable-stable angina), peritonitis, or pregnancy. A subgroup analysis was made between responders and non-responders to sauna therapy. Responders were defined as an average decrease in the degree of OH between the control and the intervention period of at least 500 ml. The study was approved by the institutional ethical committee of the Hospital Civil de Guadalajara and registered at ClinicalTrials.gov (NCT03563898). The study was conducted in accordance with the Declaration of Helsinki and follows the CONSORT specifications.

### Study period

The study was carried out in two intervals, the control and the intervention period. The control period consisted of observation for 10 days, where characteristics of the PD status, vital signs, bioimpedance spectroscopy (BIA) data, and questionnaires were recorded. Patients were weighed wearing underwear. Blood samples for chemistry, electrolytes, and blood count were collected. Blood pressure was taken daily after a 10-min rest. Questionnaire about symptoms of FO (NYHA Functional Classification of breathlessness), sleep quality scale, and antihypertensive regimens were applied to each patient. FO measurement by BIA was performed on the first day, and also daily body weight. PD treatment (volume infused, dextrose concentration, ultrafiltrate volume, and the number of exchanges per day) was recorded. A graduated glass with measurements in milliliters was provided, and the patients were instructed to record the amount of fluids ingested daily. A 24-h urine sample was collected on day one. In this phase, patients did not use the portable sauna bath.

**Figure 1:**
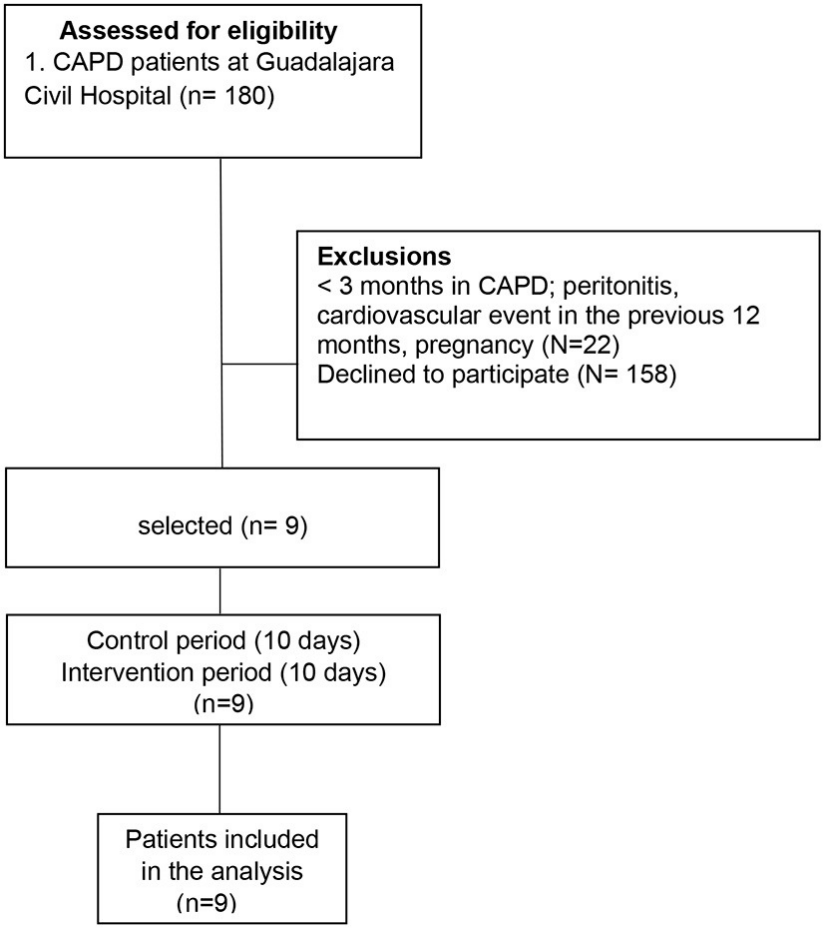
Portable sauna bath.

During the intervention period, patients used the sauna bath daily, 30 min at 45°C, sitting on a chair, for 10 consecutive days (Figure 1). Time and/or temperature of the bath was reduced according to patient's tolerance. Ten minutes before taking the sauna bath, blood pressure and weight were recorded. No special recommendation on fluid intake was made. Nephrology staff was present during the bath. Patients informed of symptoms that occurred during the intervention. After finishing the bath, they stayed 15 min to dry the body with a towel. The wet catheter dressing was changed to a dry one impregnated with mupirocin spray at the exit site, and the patient's weigth and blood pressure were recorded. During the intervention period, the same information as in the control phase was collected.

### Outcomes

The primary outcome was treatment-related adverse events (grade two or above) as measured by CTCAE v 4.0. Secondary outcomes were the degree of OH (measured by BIA), decrease in patient's weight, blood pressure, blood test, FO symptoms (NYHA Functional Classification of breathlessness) and changes in sleep quality (PSQI score) between the two periods.

### Statistics

Statistical analysis was conducted using R Studio. Because this is a pilot study, no power calculation was performed. Quantitative variables were expressed as mean ± standard deviation, or as median (range), as appropriate. The Wilcoxon Signed-Rank Test was used to determine the differences in the data collected between the two periods. A two-tailed *p* <0.05 was consider statistically significant. A subgroup analysis was made between responders and non-responders to sauna therapy. A logistic regression model was built to try to identify factors associated to non-respond.

## Results

**Figure 2:**
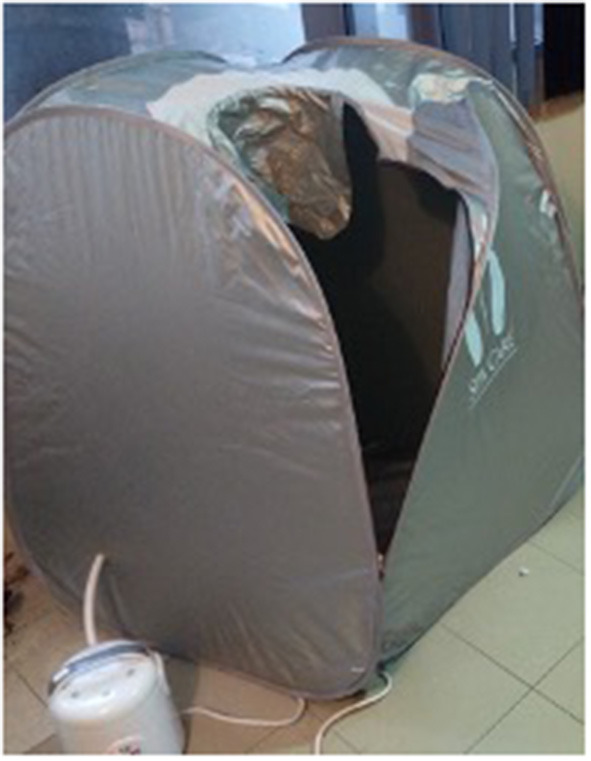
Cohort selection. CAPD, continuous ambulatory peritoneal dialysis.

Among the 180 patients dialyzed on our CAPD program, we excluded 158 because they had < 3 months in CAPD, they had peritonitis or a cardiovascular event in the last 12 months, or declined to participate in the study. Nine patients fulfilled the inclusion criteria and all were included in the analysis, (Figure 2). No patients interrupted the treatment during the study period. Patient's clinical baseline characteristics are shown in Table 1. The mean age was 36 (23–68) years, 77% were females, one-third were diabetic, and only two had diuresis > 500 ml/24 h; the remaining seven patients were considered anuric (urine outout < 100 ml/24 h); baseline systolic and dyastolic blood pressure was 147 ± 10 mmHg and 93 ± 12.3 mmHg, respectively. The overhydration index was 6.3 (2.7–15.3) L, and they had a dialysis vintage of 25 (5–72) months; more than half were using a 1.5% dextrose concentration PD fluid. All participants were hypertensive and only two were not using diuretics.

**Table 1:** Patient clinical characteristics.

	***n*** **= 9**
Age, years (range)	36 (23–68)
Female sex (%)	7 (77)
Diabetes (%)	3 (33)
Hypertension (%)	9 (100)
Body mass index, kg/m^2^ (range)	26 (21–38)
Diabetics (%)	3 (33)
Glomerular filtration rate, mean (ml/min/1.73 m^2^)	3.6
Diuresis > 500 ml/24 h (%)	2 (23)
Using diuretics (%)	5 (55)
Furosemide daily dose (mean)	120 mg
Systolic blood pressure (mmHg)	147 ± 10
Dyastolic blood pressure (mmHg)	93 ± 12.3
Overhydration index, L^*^ (range)	6.3 (2.7–15.3)
Dialysis vintage, months (range)	25 (5–72)
Number of dialysis exchanges/day	4
Dialysis glucose concentration (%)	
1.5%	5 (56)
2.5%	3 (33)
4.25%	1 (11)

Values expressed as mean and min-max.

* Bioimpedance spectroscopy.

Fifty-one of the 54 total sauna bath sessions were well tolerated; in three (5.5%) sessions adverse events were reported (Table 2). Two patients rerported momentary dizziness without hypotension at the first session, 20 min after being on the sauna bath, and these events were considered mild. These two patients received 20 min of of therapy in subsequent sessions, without any adverse effect. A patient with advanced diabetic neuropathy presented a second-degree burn, that was treated with topical analgesics and antibiotics, event that was considered moderate.

**Table 2:** Adverse events per total sauna baths sessions.

	***n*** **= 54**
Adverse events per total sauna baths (%)	
Dry skin	0
Burn	1 (1.8)
Pruritus	0
Dehydration	0
Catheter infection	0
Hypotension	0
Dizziness	2 (3.7)
Adverse events	3 (5.5)

A significant difference was observed in the degree of OH measured by bioimpedance between the control and the intervention period (6.3 ± 1.2 L vs. 5.5 ± 1.3 L, *p* = 0.05). A significant decrease in mean body weight between the two periods was observed (67.7 ± 11.4 kg vs. 66.8 ± 3.8 kg, *p* = 0.003). Individual weight losses are shown in Table 3. Additionally, there was a significant decrease in diastolic blood pressure (92 ± 13.5 mmHg vs. 83 ± 13.3 mmHg, *p* = 0.003), but not in the systolic blood pressure (148 ± 12 mmHg vs. 144 ± 16 mmHg, *p* = 0.42). An improvement in sleep quality was observed, mainly in a decrease in nocturnal awakenings and in time of falling asleep at bedtime between the two periods (Global PSQI score, 7.3 ± 3.7 vs. 5.1 ± 3.2, *P* = 0.02). There was no significant difference in the ultrafiltrate or the water intake between the two periods. The summary of anthropomorphic parameters and blood tests during the two periods are shown in Table 4. We applied the Subjective Global Assessment of nutritional status in all patients on the first day of the protocol. Seven of them had normal nutritional status and two moderately malnourished. Table 5 summarizes the patient's nutritional status and their relationship with FO, time on dialysis, albumin, and C- reactive protein.

**Table 3:** Weight loss per patient per sauna session.

	BSA^*^	**Day 1**	**Day 2**	**Day 3**	**Day 4**	**Day 5**	**Day 6**	**Day 7**	**Day 8**	**Day 9**	**Day 10**
Patient 1	1.52 m^2^	200 g	100 g	200 g	100 g	0 g	100 g	300 g	100 g	0 g	200 g
Patient 2	1.71 m^2^	300 g	300 g	300 g	250 g	350 g	700 g	300 g	200 g	100 g	100 g
Patient 3	1.74 m^2^	400 g	350 g	450 g	500 g	100 g	700 g	400 g	200 g	200 g	300 g
Patient 4	1.85 m^2^	1200 g	300 g	0 g	100 g	100 g	100 g	300 g	0 g	400 g	100 g
Patient 5	1.72 m^2^	0 g	200 g	200 g	200 g	100 g	500 g	100 g	200 g	100 g	200 g
Patient 6	1.69 m^2^	100 g	0 g	200 g	100 g	200 g	300 g	100 g	300 g	300 g	200 g
Patient 7	1.70 m^2^	200 g	100 g	500 g	200 g	300 g	200 g	300 g	100 g	500 g	200 g
Patient 8	1.84 m^2^	700 g	300 g	100 g	400 g	400 g	0 g	400 g	800 g	400 g	300 g
Patient 9	1.75 m^2^	200 g	100 g	300 g	100 g	250 g	100 g	300 g	100 g	400 g	350 g

* BSA, body surface area.

**Table 4:** Anthropomorphic parameters and electrolytes during the control and the intervention period.

	**Control period**	**Intervention period**		**Change**	**CI 95%**	***P*** -value ^*^
Ultrafiltered by dialysis (ml)	880 ± 121 (270; 1405)	860 ± 98 (321; 1323)		20 ± 35	(−62 to 102)	0.91
Extracellular water (L)	20 ± 5.1 (14.9; 30.7)	19.4 ± 4.8 (14.1; 28.4)		0.5 ± 0.5	(−0.7 to 1.9)	0.37
Intracellular water (L)	18.1 ± 3.4 (14.4; 22.3)	17.7 ± 3.3 (13.4; 23.9)		0.3 ± 0.6	(−1.1 to 1.7)	0.59
E/I (L)	1.1 ± 0.3 (0.83; 1.26)	1 ± 0.2 (0.71; 1.33)		0.006 ± 0.2	(−0.04 to 0.06)	0.95
Overhydration OH (L)	6.3 ± 1.2 (2–7; 15.3)	5.5 ± 1.3 (0.7; 14.2)		0.7 ± 0.2	(0.09 to 1.3)	0.05^*^
OH/ECW (%)	29 ± 3 (16; 50)	26 ± 4 (0.04; 50)		3 ± 1	(0 to 0.06)	1.00
Mean body weight (kg)	67.7 ± 11.4 (64; 92)	66.8 ± 3.8 (47; 91)		0.85 ± 0.1	(0.4 to 1.2)	0.003^*^
Mean body weight before-after sauna bath (kg)		Before sauna	After sauna			
		67.1 ± 3.8 (47.5; 91.8)	66.8 ± 3.8 (47.4; 91.5)	0.25 ± 0.3	(0.18 to 0.32)	0.008^*^
Mean SBP (mmHg)	148 ± 12 (135; 163)	144 ± 16 (122; 176)		3.6 ± 4.5	(−6 to 14)	0.42
Mean DBP (mmHg)	92 ± 13.5 (80; 110)	83 ± 13.3 (69; 106)		8.6 ± 2.4	(3 to 14)	0.003^*^
Mean difference SBP before-after sauna bath		Before sauna	After sauna			
		149 ± 16 (122; 171)	140 ± 22 (96; 176)	9.1 ± 4.4	(−1 to 19)	0.05^*^
Mean difference DBP before-after sauna bath		Before sauna	After sauna			
		92 ± 15 (71; 116)	79 ± 13 (69; 106)	12.9 ± 4.9	(1.4 to 24)	0.01^*^
Water intake per day (ml)	506 ± 167 (156; 733)	517 ± 187 (178; 800)		11 ± 34	(−91 to 68)	0.67
Global PSQI score	7.3 ± 3.7 (3; 14)	5.1 ± 3.2 (1; 10)		2.2 ± 0.6	(0.7 to 3.6)	0.02^*^
NYHA scale	1.8 ± 0.3 (1; 2)	1.8 ± 0.3 (1; 2)		NA	NA	NA
Hemoglobin (g/dl)	9 ± 0.5 (6.1; 12.1)	9.2 ± 0.6 (5.8; 12.3)		0.1 ± 0.1	(−0.4 to 0.1)	0.19
Hematocrite (%)	27.8 ± 1.6 (20.3; 35.2)	28.8 ± 1.9 (18.8; 36.7)		1 ± 0.4	(−2.1 to 0.02)	0.9
Urea (mg/dl)	158 ± 7 (117; 186)	159 ± 12 (111; 210)		0.9 ± 7.6	(−18 to 16)	0.9
Creatinine (mg/dl)	13.7 ± 1.2 (8.3; 20.4)	13.6 ± 1.1 (9.5; 18.3)		0.08 ± 0.41	(−0.8 to 1)	0.83
Albumin (g/dl)	3.2 ± 0.1 (2.4; 4)	3.4 ± 0.1 (2.7; 4.2)		0.2 ± 0.03	(−0.3 to −0.1)	0.9
Potassium (mEq/L)	4.7 ± 0.2 (3.7; 5.6)	4.4 ± 0.2 (3.5; 5.6)		0.2 ± 0.12	(−0.03 to 0.56)	0.07
Sodium (mEq/L)	136.8 ± 0.7 (133; 140)	137.7 ± 0.6 (134; 140)		0.8 ± 0.5	(−2 to 0.4)	0.15
Phosphorus (mEq/L)	6.2 ± 0.4 (3.5; 7.9)	6.4 ± 0.5 (3.3; 8.6)		0.2 ± 0.1	(−0.6 to 0.1)	0.22
Uric acid (mg/dl)	6.4 ± 0.3 (4.9; 8.8)	6.4 ± 0.4 (4.8; 9.1)		0.1 ± 0.2	(−0.8 to 0.5)	0.69

* Two-sided t-test p < 0.05.

SBP, Systolic blood pressure; DBP, Diastolic blood pressure; NYHA, New York Heart Association; PSQI, Sleep Quality Assessment score; OH/ECW, Over Hydratation/ ECW extracellular water; E/I, extracellular Intracellular ratio.

**Table 5:** Nutritional status by SGA.

	***n*** **= 9**
Normal nutritional status (%)	7 (77)
Moderate protein-energy wasting (%)	2 (23)
Severe protein-energy wasting (%)	0
Mean OH index in normal nutritional status (Liters)	6.7 ± 1.2
Mean OH index in moderate protein-energy wasting (Liters)	6.5 ± 1.3
Mean CRP in normal nutritional status (mg/L)	1 ± 0.6
Mean CRP in moderate protein-energy wasting (mg/L)	3.7 ± 0.9
Mean serum albumin in normal nutritional status (g/dl)	2.9 ± 1.1
Mean serum albumin in moderate protein-energy wasting (g/dl)	3.7 ± 1.5
Mean dialysis vintage (months) in normal nutritional status	41 ± 15
Mean dialysis vintage (months) in moderate protein-energy wasting	35 ± 11

OH, overhydration; CRP, C reactive protein.

In a subgroup analysis between responders and non-responders to therapy, six patients were classified as responders, and three as non-responders. Only the presence of moderate malnutrition was associated with an increased risk of being a non-responder (OR 2.14, 95% CI 1.40–3.26, *p* = 0.03) in the univariate and multivariate logistic regression model (Table 6).

**Table 6:** Logistic regression model to identify non-responders to sauna treatment.

	**Non-responders**			
	**Univariate**	***p*** **-value**	**Multivariate**	***p*** **-value**
Age (years)	1.01 (0.99–1.04)	0.16	1.02 (1.02–1.02)	0.01
Sex (male)	0.60 (0.31–1.15)	0.17	0.93 (0.91–0.96)	0.12
Diabetic	1 (0.47–2.09)	1	0.91 (0.89–0.93)	0.09
Months in CAPD	1.01 (1.00–1.02)	0.05	0.99 (0.99–1.00)	0.97
BMI (kg/m^2^)	1.01 (0.94–1.09)	0.65	0.98 (0.97–0.98)	0.03
Moderate/severe malnutrition	2.35 (1.35–4.08)	0.01	2.37 (2.31–2.42)	0.008
Dialysis ultrafiltration	0.99 (0.99–1.00)	0.31	0.99 (0.99–0.99)	0.02

CAPD, continuous ambulatory peritoneal dialysis; BMI, body mass index.

## Discussion

In this-single center, clinical pilot study, the use of a portable sauna bath in CAPD FO patients to stimulate diaphoresis was safe, and resulted in a significant decrease in FO, diastolic blood pressure and body weight. It could be safe to use in patients on DP, however, surveillance and grooming measures must be taken by the patient, and its use should be reconsidered in patients whose sensitivity is decreased, as it was the case in the patient with advanced diabetic neuropathy.

We found a significant decrease in OH, with a mean reduction of 0.7 ± 0.2 L. The decrease in FO was similar to the results of daily sauna baths in eight patients reported by Snyder and Merrill (17) in 1966 and Pruijm in 2013 (20). In our study, average weight loss per sauna bath during 20 to 30 min/day, was 250 ± 300 g. The total amount of sweat loss varied with some patients reaching 100 to 550 g, while other studies reported losses of up to 1,430 g (17). This suggests that some persons are more responsive to stimulated sweating therapies than others, explained in part by the difference in body surface area. Indeed. Although not statistically significant, we were able to observe a larger BSA in patients who responded to the sauna bath vs. non-responders (1.75 ± 0.1 m^2^ vs. 1.64 ± 0.1 m^2^, *p* = 0.06), suggesting that the larger the BSA, the greater the number of functioning sweat glands. We could identify that the presence of moderate/severe malnutrition, is a risk factor that reduces the response to fluid loss with the use of sauna bath (Table 5). Although hot weather plays a role in sweating, we do not think it influenced our results. The study was carried out during the months of January to March, when the maximum temperature in Guadalajara ranged between 24°C and 28°C.

We observed that diastolic blood pressure also presented a significant decrease before and after the therapy. This could be explained by a decrease in FO. However, this effect may be only a reflection of the heat-mediated vasodilatation of the sauna, so a larger study is necessary in which the patients be exposed to more days to therapy to confirm our hypothesis.

An improvement in sleep quality was also observed, mainly in a decrease in nocturnal awakenings and in time of falling asleep at bedtime between the two periods, using the Global PSQI score (Sleep Quality Assessment score), with a mean change of two points in the score. This result could positively contribute to the quality of life of patients. Sauna bathing could generate a gratifying and relaxing sensation, a fact that should be considered. It is possible that the good tolerance to sauna-bath stimulated diaphoresis in our population was related to our younger PD population; however, tolerance could be different in older patients.

No changes in electrolytes and urea were observed, perhaps explained by the short time of the study period. Water ingestion per day was not different between the control and the intervention period (506 ± 167 vs. 517 ± 187, respectively), which suggests that the use of sauna bathing did not cause more thirst in these patients.

Keller et al. described how perspiration has been of historical interest in the management of chronic kidney disease, due to its ability to eliminate fluids, electrolytes, and uremic toxins through the sweat glands, as well as the potential risks of exposure to heat in the sauna, such as burns and hypovolemia; despite its biological rationale, there have been only seven studies with a total of 60 patients who have explored perspiration in end stage kidney disease (21).

Our study has several limitations. The number of participants was small. The duration of the study period was short, with an exposure to therapy for only 10 days. It was a pilot study and was performed in a single center. We did not evaluate the urea and potassium kinetics, solutes that may have changed during the intervention but were not considered as objective of our study. The absence of a control group.The study was carried out in a selected group of PD patients, which may not be representative of the entire PD population, Finally, the conditions of the sauna bath sessions, such as temperature and duration, were established based on the capabilities set by the manufacturer of the equipment. We do not rule out that other parameters could be evaluated in these patients to make it more effective and tailored to the tolerance for each patient. In our opinion, patients could undergo two sauna bath sessions per day since it was very well tolerated. However, we did not have enough personnel to supervise more than one sauna session per day.

In conclusion, in PD patients with FO, stimulated diaphoresis with sauna baths can be an effective, safe and sustained way to reduce FO. However, surveillance and grooming measures must be taken by the patient, and its use should be avoided in patients whose sensitivity is decreased, as it was the case in our patient with advanced diabetic neuropathy.

We believe that more studies are needed to evaluate a longer time the use of the sauna bath in a certain type of patient on PD or HD, especially the those with anuria and poor ultrafiltrate.

## Data availability statement

The original contributions presented in the study are included in the article/supplementary materials, further inquiries can be directed to the corresponding author.

## Ethics statement

The studies involving human participants were reviewed and approved by Comité de Etica e Investigacion, Hospital Civil de Guadalajara Fray Antonio Alcalde. The patients/participants provided their written informed consent to participate in this study.

## Author contributions

PM-A, JC-I, and GG-G were responsible for the conception and design of the work, the acquisition, analysis, and interpretation of data, drafting the work, and approving the version for publication and are accountable for the aspects of the study that this entailed. PM-A and JC-I were responsible for the analyses and interpretation of data, critical revision of the draft for important intellectual concepts, and approving the version for publication. GN-B, KH-M, AG-O, LA-V, OM-M, and VL-A were responsible for the acquisition of data and final approval of the version for publication and are accountable for the aspects of the study that this entailed. All authors contributed to the article and approved the submitted version.

## Conflict of interest

The authors declare that the research was conducted in the absence of any commercial or financial relationships that could be construed as a potential conflict of interest.

## Publisher's note

All claims expressed in this article are solely those of the authors and do not necessarily represent those of their affiliated organizations, or those of the publisher, the editors and the reviewers. Any product that may be evaluated in this article, or claim that may be made by its manufacturer, is not guaranteed or endorsed by the publisher.

## References

[1] Blake PG Bargman JM Brimble KS Davison SN Hirsch D McCormick BB. Clinical practice guidelines and recommendations on peritoneal dialysis adequacy 2011. Perit Dial Int. (2011) 31:218–39. 10.3747/pdi.2011.00026 21427259

[2] Wabel P Moissl U Chamney P Jirka T Machek P Ponce P. Towards improved cardiovascular management: the necessity of combining blood pressure and fluid overload. Nephrol Dial Transplant. (2008) 23:2965–71. 10.1093/ndt/gfn228 18458032

[3] Chazot C Wabel P Chamney P Moissl U Wieskotten S Wizemann V. Importance of normohydration for the long term survival of haemodialysis patients. Nephrol Dial Transplant. (2012) 27:2404–10. 10.1093/ndt/gfr678 22253067

[4] Agarwal R. Hypervolemia is associated with increased mortality among hemodialysis patients. Hypertension. (2010) 56:512–7. 10.1161/HYPERTENSIONAHA.110.154815 20625076 PMC2929660

[5] Paniagua R Ventura M Avila-Díaz M Hinojosa-Heredia H Méndez-Durán A Cueto-Manzano A. NTproBNP, fluid volume overload and dialysis modality are independent predictors of mortality in ESRD patients. Nephrol Dial Transplant. (2010) 25:551–7. 10.1093/ndt/gfp395 19679559

[6] Tzamaloukas AH Saddler M Murata GH Malhotra D Sena P Simon D. Symptomatic fluid retention in patients on continuous peritoneal dialysis. J Am Soc Nephrol. (1995) 6:198–206. 10.1681/ASN.V62198 7579085

[7] Wizemann V Wabel P Chamney P Zaluska W Moissl U Rode C. The mortality risk of overhydration in haemodialysis patients. Nephrol Dial Transplant. (2009) 24:1574–9. 10.1093/ndt/gfn707 19131355 PMC2668965

[8] Demirci MS1 DC. Relations between malnutrition-infl ammationatherosclerosis and volume status. The usefulness of bioimpedance analysis in peritoneal dialysis patients. Nephrol Dial Transplant. (2011) 26:1708–16. 10.1093/ndt/gfq588 20921295

[9] Foley RN Herzog CA Collins AJ. United States renal data system. Blood pressure and long-term mortality in United States hemodialysis patients: USRDS waves 3 and 4 study. Kidney Int. (2002) 62:1784–90. 10.1046/j.1523-1755.2002.00636.x 12371980

[10] Kalantar-Zadeh K Regidor D Kovesdy CP Van Wyck D Bunnapradist S Horwich TB. Fluid retention is associated with cardiovascular mortality in patients undergoing long-term hemodialysis. Circulation. (2009) 119:671–9. 10.1161/CIRCULATIONAHA.108.807362 19171851 PMC2773290

[11] Kimmel PL Varela MP Peterson RA Weihs KL Simmens SJ Alleyne S. Interdialytic weight gain and survival in hemodialysis patients: effects of duration of ESRD and diabetes mellitus. Kidney Int. (2000) 57:1141–51. 10.1046/j.1523-1755.2000.00941.x 10720966

[12] Saran R Bragg-Gresham J Rayner HC Goodkin DA Keen ML Van Dijk PC. Nonadherence in hemodialysis: associations with mortality, hospitalization, and practice patterns in the DOPPS. Kidney Int. (2003) 64:254–62. 10.1046/j.1523-1755.2003.00064.x 12787417

[13] Charra B. Fluid balance, dry weight, and blood pressure in dialysis. Hemodial Int. (2007) 11:21–31. 10.1111/j.1542-4758.2007.00148.x 17257351

[14] Kim Y Biesen WV. Fluid overload in peritoneal dialysis patients. Semin Nephrol. (2017) 37:43–53. 10.1016/j.semnephrol.2016.10.006 28153194

[15] Lacher JW Schrier RW. Sweating treatment for chronic renal failure. Nephron. (1978) 21:255–9. 10.1159/000181401 714199

[16] Man in 't Veld AJ van Maanen JH Schicht IM. Stimulated sweating in chronic renal failure. Br Med J. (1978) 2:172–3. 10.1136/bmj.2.6131.172-a 678833 PMC1606288

[17] Snyder D Merrill JP. Sauna baths in the treatment of chronic renal failure. Trans Am Soc Artif Intern Organs. (1966) 12:188–92. 5960700

[18] Shibasaki M Crandall CG. Mechanisms and controllers of eccrine sweating in humans. Front Biosci. (2010) 2:685–96. 10.2741/s94 20036977 PMC2866164

[19] Cui CY Schlessinger D. Eccrine sweat gland development and sweat secretion. Exp Dermatol. (2015) 24:644–50. 10.1111/exd.12773 26014472 PMC5508982

[20] Pruijm M El-Housseini Y Mahfoudh H Jarraya F Hachicha J Teta D. Stimulated sweating as a therapy to reduce interdialytic weight gain and improve potassium balance in chronic hemodialysis patients: a pilot study. Hemodial Int. (2013) 17:240–8. 10.1111/j.1542-4758.2012.00751.x 23013432

[21] Keller RW Jr Kopple JD Kalantar-Zadeh K. Perspiration interventions for conservative management of kidney disease and uremia. Curr Opin Nephrol Hypertens. (2020) 29:57–63. 10.1097/MNH.0000000000000569 31743242

